# A National Audit of Mammography Systems Settings That May Affect the Output of Artificial Intelligence Software †

**DOI:** 10.3390/diagnostics16121842

**Published:** 2026-06-14

**Authors:** Alistair Mackenzie, John Loveland, Leila Farshadi, Carlijn Roozemond, Ruben E. van Engen

**Affiliations:** 1 Medical Physics Department, Royal Surrey NHS Foundation Trust, Guildford GU2 7XX, UK; john.loveland@nhs.net (J.L.); l.farshadi@nhs.net (L.F.); 2 Diagnostic Radiology Physics, Maidstone and Tunbridge Wells NHS Trust, Maidstone ME16 9QQ, UK; 3 Radiological Sciences Unit, Imperial College Healthcare NHS Trust, London W6 8RF, UK; 4 Dutch Expert Centre for Screening (LRCB), Wijchenseweg 101, 6538 SW Nijmegen, The Netherlands; c.roozemond@lrcb.nl (C.R.); r.vanengen@lrcb.nl (R.E.v.E.)

**Keywords:** Artificial Intelligence, image processing, mammography, DICOM

## Abstract

**Background:** Artificial Intelligence (AI) software in mammography is trained on a set of processed images and may be less effective when applied to images acquired on different systems or systems with different processing and/or acquisition settings. The aim of this work was to undertake a retrospective audit of a large number of mammography systems in the United Kingdom and identify the number of differences in image acquisition and processing factors. **Methods:** Images of the TORMAM phantom are acquired as part of the routine quality control programme. Data from the DICOM header of these images were extracted to provide a snapshot in time of the system configurations. A longitudinal audit of DICOM header data for all of the Hologic systems was tested by one medical physics department (MPD1) over 14 years. **Results:** We received results from 28 UK medical physics services for 498 systems. There were 7 different models of mammography systems, each with up to 7 different versions of acquisition workstation software. Each mammographic model had multiple image processing versions, including bespoke settings. The GE had two dose settings, while Siemens systems had a range of doses from 80% to 150% of the standard dose. In the longitudinal audit, there were between 2 and 6 software versions in concurrent use on the Hologic systems tested by MPD1. **Conclusions:** This study showed the heterogeneity of system setup across the UK in a single year, as well as changes to system setup over time. These differences may affect the outcomes of both AI and human readers. There are responsibilities on AI suppliers, mammography equipment manufacturers, breast-screening units, and medical physics services to ensure outcomes are not adversely affected by differences or changes in mammography equipment configurations.

## 1. Introduction

The introduction of Artificial Intelligence (AI) as part of breast screening programmes has already begun and can be implemented as a reading aid for the radiologist, as a triage tool to distinguish between low and high-risk cases, or to completely replace human readers [1,2,3,4]. AI algorithms have also shown promising results in risk prediction, density estimation and quality control [5,6]. Whilst AI has the potential to improve the outcomes of breast cancer screening and/or aid more efficient workflows, it carries risks if it is incorrectly implemented or is biased [7]. Many of the factors that can affect the outcome of AI are unknown, but it has been shown that detectability varies with image appearance and cancer characteristics [8,9].

AI companies currently design products to work with processed ‘FOR PRESENTATION’ images largely because unprocessed mammography data are rarely stored long-term and are thus less readily obtained. The processing applied to mammography images might involve noise reduction, edge enhancement, frequency processing, contrast adaptation to better match the visual response of the eye, as well as more sophisticated content- based adjustments. The processing attempts to optimise the appearance of the image for the human viewer; however, some information is likely to be lost or become less visible. It has been shown that image processing can affect the underlying sharpness and noise of images [10]. Structures might appear differently depending on the applied processing techniques, and so it is unlikely that all current processing is optimal for AI software. The training of AI needs to account not just for differences in acquisition, but also for different versions of image processing.

Many screening centres have an image processing setup using the default possibilities offered by the vendor. For most systems, this means that centres can choose up to six different image perception tastes, each with a distinct and notably different appearance. Some vendors, however, do the tuning of their processing on-site, and for these systems, it is unlikely that all centres will have similar image processing settings. The supplier can also adjust image processing to suit a breast screening centre’s requirements, but this may result in non-optimal presentation of images. The personal experience of the authors in official nationwide type testing is that some image processing packages/settings could be improved.

It has been shown that image processing affects the decision-making of human readers, in particular for the task of detecting lesions that should be recalled for further investigation [11,12]. However, can AI products still perform as expected if image processing is changed, i.e., is the AI generalisable over a range of appearances? A retrospective AI study [13] of screening images found that the AI began recalling approximately four times more cases than previously following an image processing software upgrade that changed the sharpness of the images, but was hardly visible to the human eye. After readjustment of the operating point for the AI, the recall rate dropped again to normal levels. This suggests that other factors impacting the visual appearance of images or statistical distributions within the image data may have a similar impact on AI reader performance.

Beyond processing effects, image features can be affected by the software and hardware used, the dose level used, changes to the detector design or detector readout, detector ageing, the use and type of anti-scatter grid or the type of compression paddle. These factors could lead to differences ranging from noise characteristics or sharpness to a difference in the distribution of the white and dark areas in clinical images. As AI software operates as a black box, it is not known which image features influence the AI decision. A number of researchers have tested the effect of changing the image presentation and/or the training set [14,15,16,17] and found differences in the outcome of the AI when applied to datasets acquired under different conditions, e.g., manufacturer, dose level. Such differences could potentially complicate the rollout of AI products on a national scale. It is important to recognise that there might be differences between the local clinical images and the training dataset of the AI software.

The training of AI requires the collection of large numbers of images with their clinical outcomes. Ideally, the training dataset will be recent and from a wide range of cases and settings. However, in practice, the dataset will be at least a few years old due to the collection time required and the ideal criterion that an image can only be considered normal after a follow-up screening event. In this context, we need to appreciate that mammography systems will be upgraded, settings will be changed, and systems or parts will be replaced over time. This means that local image characteristics could increasingly deviate from training datasets.

This article is a revised and updated version of a proceeding entitled “Survey of image processing settings used for mammography systems in the United Kingdom: how variable is it?”, which was presented at IWBI, Chicago, 2024 [18]. The data were re-analysed, and the work was expanded to include more data.

There is good evidence in the literature that image presentation can impact the effectiveness of AI models, especially if the characteristics of the training set differ from the local clinical images. However, it is not clear what differences already exist in the clinical environment. Therefore, the main aim of this work was to undertake a retrospective audit of a large number of systems used in the United Kingdom breast screening centres and identify the heterogeneity of the setups of the systems in terms of image processing, image acquisition, and hardware differences (e.g., anti-scatter grids). These parameters may influence the presentation of the clinical images and potentially the performance of AI software. The second aim was to examine the image processing settings over time for a limited number of systems. If the different perception of images influences the outcome of AI software, it is a key piece of knowledge to gain an understanding of the timescales that image processing settings are updated and what these updates might include.

## 2. Materials and Methods

### 2.1. Data Extraction Tools

Mammography images are stored in Digital Imaging and Communication in Medicine (DICOM) format. The image will have a header that contains information including the patient’s details, how the image was acquired, and the image processing version and settings. The data are stored in a series of tags, corresponding to different codes. Most of these codes are described in the DICOM standard [19], but some tags are reserved as private tags for the manufacturer to use. A limited amount of information about the private tags may be found within the manufacturer’s DICOM conformance statements. In practice, different DICOM tags can be used for nominally equivalent data on system settings by each manufacturer. The tags relevant to this study were chosen by reading these standards, conformance statements and obtaining information from individual manufacturers. An initial set of ninety-three DICOM tags was selected, which specified the mammography system, or those relating to the image acquisition and image processing. Due to the uncertainties in the identification of tags related to the perception of images, more tags were initially considered than were expected to be used for this study. After investigation of the contents of all initial ninety-three tags for the four manufacturers, the tags selected for this study were chosen and are shown in Table 1.

**Table 1:** Data extracted from DICOM headers, brand-specific information is labelled (GE), (Ho) and (Sm) for GE Healthcare (Buc, France), Hologic Inc (Newark, DE, USA) and Siemens Healthineers (Forchheim, Germany), respectively.

DICOM tag name	DICOM Tags ^1^
Presentation intent type	0008x, 0068x
Model name	0008x, 1090x
Institution name	0008x, 0080x
Station name	0008x, 1010x
Acquisition date	0008x, 0022x
Derivation description	0008x, 2111x
Software version	0018x, 1020x
Image processing	See Table 2
Dose level	0018x, 7062x (GE, Sm), 0019x, 1049x (Ho)
Anti-scatter grid	0019x, 109Cx
Compression paddle type	0018x, 11A4x, 0018x, 7062x

^1^ ‘x’ indicates that the tag is hexadecimal.

**Table 2:** Number of systems, number of software versions used, number of image processing versions and/or settings and the tags where the information is held.

Unit	Number	No. AWS Software Versions	No. of Image Processing Versions and/or Settings	Tag(s) ^1^ for Image Processing
Fujifilm Amulet	7	7	5	0008x, 2111x
GE Essential	19	6	3	0018x, 1400x, 0018x, 7006x
GE Pristina	116	4	3	0018x, 1400x, 0018x, 7006x
Hologic Selenia Dimensions	126	7	4	0019x, 1062x
Hologic 3Dimensions	155	4	5	0019x, 1062x
Siemens Inspiration	43	6	10	0019x, 1010x
Siemens Revelation	32	2	6	0019x, 1010x

^1^ ‘x’ indicates that the tag is hexadecimal.

### 2.2. Retrospective Audit of United Kingdom (UK) Mammography Systems

**Figure 1:**
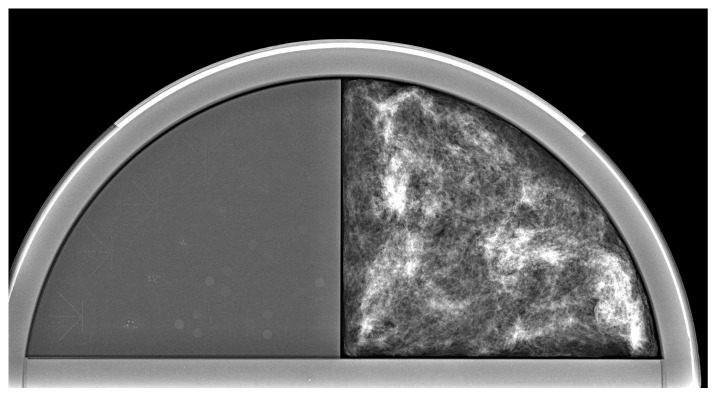
Image of the TORMAM phantom.

The use of the TORMAM phantom (Leeds Test Objects, Leeds, UK) (Figure 1) is a recommended test of overall image quality by the NHSBSP (National Health Service Breast Screening Programme) routine quality control (QC) protocol [20]. The TORMAM phantom contains various breast-like structures, and the ‘FOR PRESENTATION’ image should be scored. The authors are aware that image processing will depend on the specific object being imaged and that differences exist between the phantom and clinical mammography images. The processing may therefore function differently when applied to breast tissue compared to the test object, and the interpretation of the resulting phantom image quality should be handled with care. However, the phantom image metadata will give information on the system (settings) and may give an indication of the effect of different processing settings on clinical images.

We undertook an audit of mammography systems in the UK by collating DICOM data from TORMAM phantom images. We invited 35 medical physics departments that undertake quality control testing of UK mammography systems used in breast screening programmes and other mammography imaging departments to send header data extracted from the images of the TORMAM phantom (Figure 1). Due to notable variability in TORMAM phantom construction, we generally did not request images or their associated image quality scores from the departments, but for a few cases, we requested images for illustration purposes.

Some centres extracted data from multiple images for a system. The data were collated and filtered to only include ‘FOR PRESENTATION’ images (‘Derivation Description’ tag), the most recent image acquired (‘Acquisition Date’ tag), and full-field digital mammography, with no prone tables or biopsy add-ons. The content of the data in the tags from Table 1 was then compared between these imaging systems. The full set of anonymised extracted data can be found in the supplement for 403 of the 498 systems (for the systems we have permission to share the data).

### 2.3. Data Collection over Time from One Medical Physics Department

As one of the 35 medical physics services, the Regional Radiation Protection Service (RRPS) of the Royal Surrey NHS Foundation Trust covers several mammography imaging units from different screening units. Typically, each system is tested every six months, and at these visits, RRPS routinely archives processed images of the TORMAM phantom for reference purposes. Within this archive, it was observed that only Hologic systems had sufficient numbers for a meaningful analysis over the full range of dates. This covered 26 systems from 2011 to 2024. The data from January 2023 to February 2024 in this dataset would have been included in the national data collection described above.

An in-house tool called the DicomTagExtractor (available on https://medphys.royalsurrey.nhs.uk/nccpm/?s=breast-dose, access date: 19 February 2024) was previously designed to extract data from DICOM headers for dose audits. This programme was adapted to include the extraction of tags mentioned above. Anonymisation was not required as only data from phantom images were used. This meant that all DICOM tags could be used without the requirement to delete or change DICOM metadata before exporting.

## 3. Results

### 3.1. Audit of Image Processing Versions

As part of the audit, 28 out of 35 physics services responded and sent data from at least one system. Not all physics groups use a TORMAM phantom or keep a copy of TORMAM images, and so some were unable to take part in this audit. In total, we received data for 498 systems between January 2023 and February 2024. The split by mammography unit model is shown in Table 2.

The software version was found in tag (0018x, 1020x) for the acquisition workstation. Table 2 lists the number of software versions and image processing settings that were found in the audit for each model of mammographic unit. It also shows the DICOM tag used by each manufacturer for storing the information on the image processing, and that each manufacturer stores this in a different location in the header. Table 2 shows that each model had between 2 and 7 different acquisition workstation (AWS) software versions across the UK from January 2023 to February 2024.

### 3.2. Image Processing Versions

Table 2 shows that five different image processing settings were utilised across the seven Fujifilm Healthcare (Kanagawa, Japan) systems. There were five different breast screening units with Fujifilm systems, and each centre had different image processing settings, suggesting site-specific configurations.

There were six different processing settings found on the GE Healthcare systems: on the Essential (Premium_View (6) and PV_Medium (3), eContrast3 (10)) and on the Pristina (eContrast3 (74), eContrast4 (40), eContrast6 (1) and no info (1)). The differences in eContrast processing change the appearance of images, as can be seen in examples on the GE website [21]. The GE system also has three levels of sharpening that can be applied to the image called fineview, ‘MTFcomp2.1’ (81), ‘MTFcomp2.2’ (32) and ‘MTFcomp2.3’ (18) and four unknown.

Siemens has a number of ‘flavours’ of processing that can be set up. We know that the processing settings can not only affect the appearance of the images, but can also affect the detection of cancers by human readers [22]. In this audit, two ‘flavours’ (Flavor0 (18) and Flavor1 (30)) were in use, but there was also a Gen1 and Gen2 classification, e.g., F0_Gen1 (13), F0_Gen2 (5), F1_Gen1 (19) and F1_Gen2 (3), all of which were in current use. For the Inspiration, the image processing settings were called Contrast Levels: Standard_OV2 (5), Med (3), MedHigh (2) and High (12) and two unknown. There were also a number of underlying tags describing the image processing settings, which varied with the processing name. There was one centre that appeared to have bespoke image processing (the processing name in the DICOM tags was named after that centre) with processing parameters distinct from other systems surveyed in this study.

The Hologic tag (0019x, 1062x) is very long and contains a number of data points, which we have called subtags. The length of this tag was found to vary, with some systems containing up to 145 separate subtags, of which some describe the setup of the system, including the image processing options. Some examples of the changes seen in the software versions are seen in Table 3, the data were collated from the customer release notes from the Hologic archive [23] and the Dutch evaluation programme. This documentation demonstrates the kind of changes which can be implemented in new software versions.

### 3.3. Example Images with Different Image Processing

**Figure 2:**
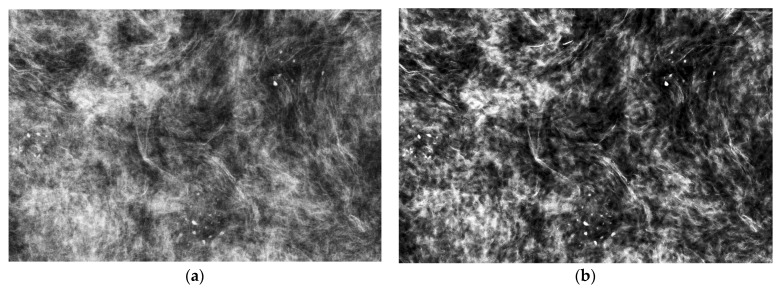
Section of TORMAM phantom acquired on the same Fujifilm Amulet system (**a**) original image (**b**) image acquired five months later, following a change in the acquisition workstation software version and image processing factors.

**Figure 3:**
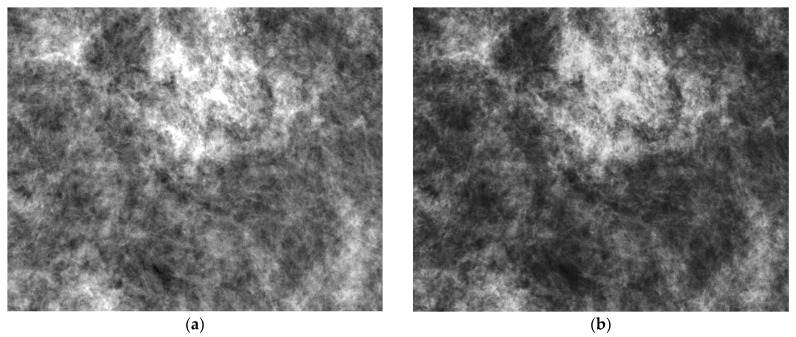
Section of TORMAM phantom acquired on two different Siemens Inspiration systems with image processing algorithms set up differently between the two breast screening centres (**a**) Standard_[*BSUname*]_Flavor; (**b**) Standard_OV2HighContract.

Figure 2 and Figure 3 show examples from two systems (Fujifilm and Siemens, respectively) acquired during routine QC using NHSBSP protocols [20]. The values in the image processing tags are different. It is normally difficult to compare TORMAM images due to variability in the phantom construction. Crucially, a visual comparison between the images was possible here as the same TORMAM was used. The TORMAM images have notably distinct contrast and grey value differences in the materials simulating the glandular breast tissue.

Figure 2 shows two images of the same TORMAM phantom acquired on the same Fujifilm Amulet system, but the image processing has been updated between tests. The software version was also changed from V9.3.2174 to V9.3.6356.0002 and the processing settings from G2.1t#0.79-0.13, MCF1.5EI1.1, C*1.0*1.0 to G1.0y#0.70+0.15, Yop2.4 gw0.750.70, C*1.0*1.0. The acquisition parameters were comparable (Figure 2a) 28 kV, W/Rh anode/filter combination, 95 mAs and (Figure 2b) 29 kV, W/Rh, 93 mAs, so the difference in appearance is attributed to differences in image processing. We are assuming here that the appearance of the clinical images would also differ between these processing settings; however, this was not reviewed as part of this study. From a visual examination, we observed that the latter image acquired has a higher contrast.

Figure 3 shows two images from two different Siemens Inspirations systems, both utilising software version VB41A, but with the following image processing name and settings and radiographic factors:

• Figure 3a: Standard_[*BSUname*]_Flavor: AM(1/1.84)_NI(7)_GaL(31)_H(127/0.00)_E(15/0.00/0)_AE(3/-0.30)_I(0/0.00010/-30)_WM(2/200/5.00)_VM(1)_GL((3/4.00/2048/320)), 29 kV, W/Rh, 101 mAs
• Figure 3b: Standard_OV2HighContrast: AM(1/1.81)_NI(1)_GaL(31)_H(127/0.00)_E(15/0.00/0)_AE(3/-0.30)_I(0/0.00010/-30)_WM(2/200/5.00)_VM(0)_GL((2/3.00/2048/270)), 29 kV, W/Rh, 111 mAs

The image in Figure 3a appears to have been set up specifically for that screening unit. It was named after the Breast Screening Unit (anonymised here) and had different image processing factors from any of the other Siemens systems in the UK. The underlying data can be found in the Supplementary Materials.

### 3.4. Dose Level Settings

The magnitude of the X-ray signal reaching the detector will be controlled by the automatic exposure control (AEC). There are different methods for selecting the cut-off point for an exposure and thereby the dose to the detector. The method will need to account for the radiographic factors selected, breast thickness and dense regions within the breast. Generally, there will be a default setting depending on the brand and type of system and patient breast thickness; however, some systems will have options to adjust the dose to a lower or higher value. In some systems, this can be selected manually for each exposure, and in others, this is set by the service engineer.

The GE Essential and Pristina have three dose levels, one of which is set as the default. Once the default level is set, other dose levels can still be selected manually. In this audit, none of the systems used the low dose. Most systems were set to the standard (STD) dose level (116), and a small number used the higher dose (19) (‘Contrast’ for Essential, ‘STD+’ for Pristina).

At the breast screening unit’s request, the Siemens engineer can configure the dose level for the AEC on Siemens systems. The standard dose setting is referred to as 100%, and this value is chosen to obtain a specific image quality by Siemens, i.e., the 100% dose level may vary slightly between different Siemens systems of the same model. The breast screening unit, in conjunction with its medical physics expert, can request to operate at a dose different from standard. The percentage dose level set for a system can be found in tag (0018x, 7062x), and the ranges of the dose setups are summarised in Table 4. The lowest dose for Siemens was 80%, and all of the cases with reduced dose (<100%) were acquired using PRIME (Progressive Reconstruction, Intelligently Minimising Exposure), which is a technique that is used to image breasts below a certain compressed thickness without the use of an anti-scatter grid and thus allows the use of lower doses. There is additional processing to compensate for the differences in signal due to the presence of extra scattered radiation in the image. 32% of the systems were set up at the default dose of 100%. Most of the systems were set up to give a higher dose than the standard, and even up to 50% higher. The quality of the images will be quite different; there will be relatively less noise in the highest dose images, but in clinical images, there will be more potential for movement blur due to the longer exposure times.

### 3.5. Physical Differences

Hologic changed its style of anti-scatter grid from a cellular design (HTC—High Transmission Cellular) [24] to a more standard linear grid. All 3Dimensions systems have a linear grid, and 78% of the Selenia Dimensions have an HTC grid. Potentially, this will have an influence on the noise patterns and/or contrast in the images.

There are different styles of compression paddles, such as flat, curved, flex, and FitSweet. These paddles each result in a different breast thickness profile across the breast support table. Such variations are corrected within the processing and can lead to differences in the perception of (clinical) images [25,26]. The data for the paddles were mostly found in ‘Paddle Description’ in DICOM tag (0018x, 11A4x). However, some information was stored in ‘Exposure Control Mode Description’ in DICOM tag (0018x, 7062x) as a code number between 5 and 24, with nine unique values. A wide range of paddles was used by the centres in these QC tests. For the systems with clearly defined paddle information, there were 331 that used the 18 cm × 24 cm or 19 cm × 23 cm paddles and 101 that used 24 cm × 30 cm paddles. There may be differences in the paddles used for the TORMAM QC exposures versus the clinical exposures; for example, no Hologic smart curve paddles were used for the imaging of the TORMAM, but they are likely to be in use for clinical images.

### 3.6. Longitudinal Audit of Software Versions of Hologic Systems from One Medical Physics Department

**Figure 4:**
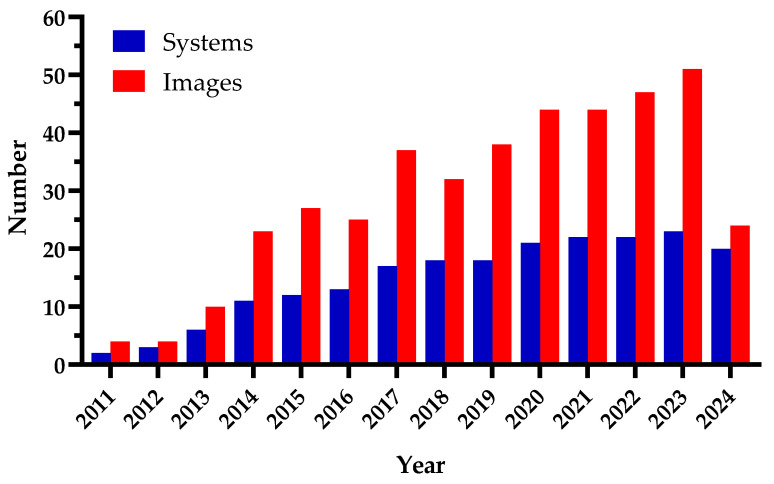
Number of Hologic systems investigated across 2011 and 2024 for one medical physics department.

**Figure 5:**
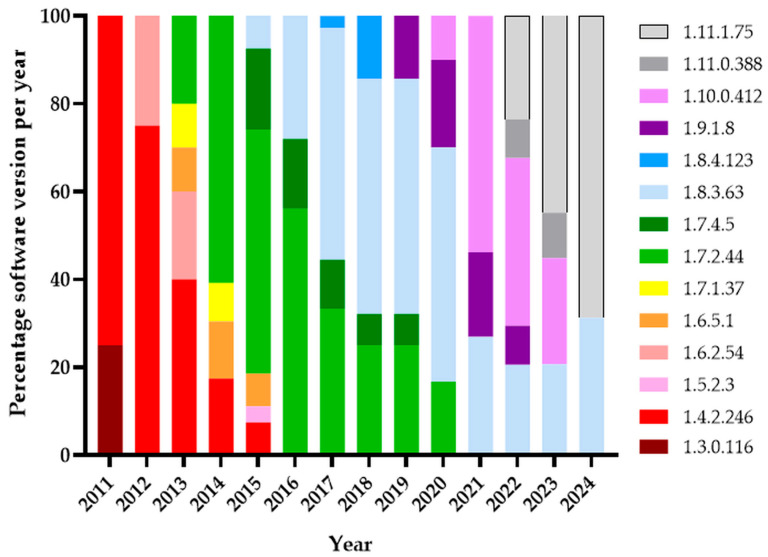
Distribution of software versions (SV) across time for Hologic Selenia Dimensions systems for one medical physics department.

**Figure 6:**
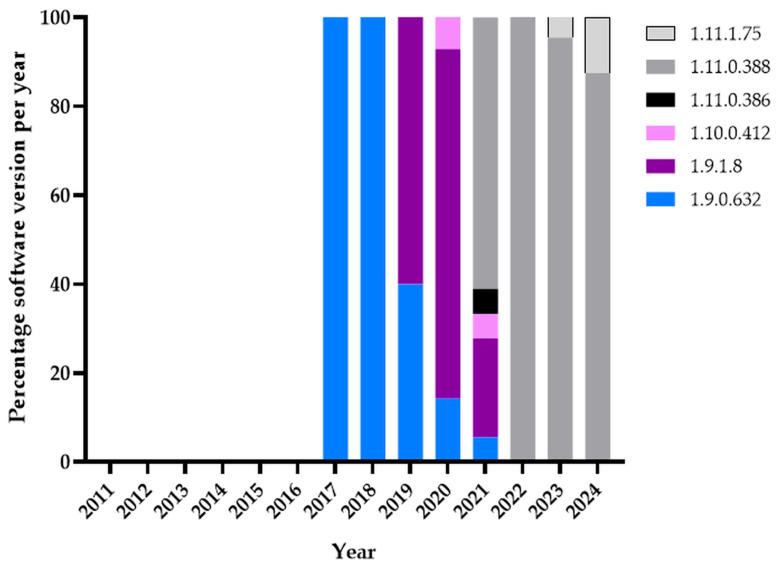
Distribution of software versions (SV) across time for Hologic 3Dimensions systems for one medical physics department. The system was first introduced in 2017.

Figure 4 shows the number of systems and TORMAM images reviewed in each year, with 2024 reflecting a reduced number as the longitudinal audit ended mid-year. There were 26 Hologic mammography systems used in the longitudinal audit (Dimensions: 17; 3Dimensions: 9), from which 410 TORMAM images were collected from 2011 to 2024 from one medical physics department. Figure 5 and Figure 6 show the distribution of software versions over time of the Hologic Selenia Dimensions and 3Dimensions, respectively. It can be seen that the number of versions in use at any one time varied between 2 and 6. A specific software version could be in use for many years; for example, version 1.8.3.63 had been used for 10 years and was still in use at the end of the audit in 2024. Some versions were only present for one or two years, e.g., 1.5.2.3 and 1.8.4.123. It is also of particular interest to note the change to version 1.10.0.412; this was the software change that caused a large increase in the number of recalls in the de Vries study [13].

Table 5 summarises the variability in image processing subtags across different AWS software versions for the 26 Hologic systems investigated within the Hologic tag (0019x, 1062x). The number of subtags within the Hologic tag increased with newer AWS software iterations, rising from 11 subtags in version 1.3.0.116 to 145 subtags in version 1.11.1.75. For every AWS software version studied, all associated TORMAM images contained an identical number of subtags. While the number of subtags remained constant within a version, the values within those subtags exhibited variability. Between 1 and 5 unique combinations of settings were identified per software group. We do not have information on whether this will affect the image perception.

## 4. Discussion

We have demonstrated the heterogeneity in the setup of mammography systems across the UK and also in software versions over time. These results are derived from an audit of DICOM header metadata and do not directly quantify differences in clinical image appearance or AI performance. The outcome of AI models will depend not only on the degree of change in the image presentation, which is not quantified here, but also on the generalisability of the AI model. However, our work does demonstrate potential differences that may need to be taken into consideration by groups who are developing AI and need to consider their training and testing datasets. Imaging departments implementing developed AI products may need to consider that the training set is likely to be older and potentially different from the setup of their equipment.

The data produced in this work from the national audit are strong, as there was a very high response rate covering 498 separate systems from 28 medical physics departments, which is most of the systems used in the UK breast cancer screening programme. The main aim of this work was to examine the setup of the systems as seen in images acquired with clinical processing. We have found a range of image processing versions, image processing settings, dose levels and differences to system components, i.e., grids and paddles, many of which we have shown to lead to differences in image perception. There are limitations on the ability to compare the effects of these differences on image quality because TORMAM phantoms have a wide variation in internal configurations and appearances and are not particularly breast-like, so they can only give an indication of the effect of changes on clinical images. However, visual inspection of some example images demonstrated that these processing differences were not only seen in the header but were also apparent in the appearance of the TORMAM images.

In the national audit, we have shown that each X-ray model had a number of different software versions that were in use across the UK at the time of the audit. Furthermore, we undertook a longitudinal audit of a single medical physics department’s data over 14 years for the Hologic systems. We showed that there were multiple software versions present at any one time, and that software versions can be in use for many years or for very short periods of time.

We do not know all of the differences between the software versions, and it is likely that some changes will not affect image processing or image presentation, but only other parts of the system. However, it is known that past software updates have included changes to: AEC calibration curves, display of implants, skin edge appearance, contrast levels, flat fielding method, metal artefact reduction, and the appearance of tissue near the skin edge. Most suppliers will produce some notes to explain the changes made between software versions, but these are not always comprehensive. Software updates that change the image presentation can lead to differences in the scores from an AI model; examples for Hologic software are shown in Table 3.

There are a number of studies that have shown that differences in the image acquisition systems and/or settings between the training set and the clinical image set can affect the AI outcome [13,14,15,16,17]. There may even be differences in AI outcome between screening units due to population differences across the country or variations in radiographic techniques, such as compression paddles [27] used and magnitude of compression, as these might change the appearance of the images. In addition, techniques such as PRIME make a correction for the estimated signal from the scattered radiation or image signal corrections to accommodate differences in local compressed breast thickness when using some compression paddles, which adds another level of processing.

The GE and Siemens systems have been set up with different dose level settings at different centres. The dose level determines the noise within the image, and there is some evidence that it can affect the outcome of an AI reader [14]. It is known that the dose level can affect the detection of subtle calcification clusters by human readers [28].

Some of the differences described are due to updates of the equipment; however, some are due to user preferences such as processing parameters or dose levels. All of these differences in image appearance are likely to affect the implementation and continued use of AI in mammography screening units. It seems vital that there is some process for type testing or acceptance testing of any new AI product, in particular, its robustness. The appearance of images used for the initial training of AI may not be the same as used in a screening unit; indeed, it is quite possible that training was undertaken on images acquired on systems with software versions/image processing that are no longer in use clinically. In practice, the introduction of AI might require a local training set, but it is unlikely that a centre will have sufficient cases which are annotated and have a ‘gold’ truth to retrain or fine-tune AI software; however, such a set could be used to adjust the operating point of the AI. Systems offering a range of image appearances will be a challenge to developers of AI models. Therefore, it seems unlikely that a trained AI will work identically and optimally across all breast screening units.

The information from the longitudinal audit showed that updates can be made to the systems that will affect the image presentation, such as changes to image processing settings or dose levels. Physical changes such as a new detector or grid may also affect the image quality and image perception. Close co-operation between breast screening services, the Medical Physics Expert and the manufacturer is essential when implementing changes. Therefore, it is essential that the AI is not only tested at installation, including performance and robustness evaluation, but also monitored throughout the lifetime of the product. Caution regarding changes in image characteristics is required, as changes to systems such as software versions can sometimes not be reversed if it is found that the update has adversely affected the effectiveness of the AI.

There is a strong argument that training the AI software should be undertaken using ‘FOR PROCESSING’ images. There will be potentially more information in the images, and fewer changes to the appearance of these images will happen. Unfortunately, it is not common for this type of image to be stored, and so there would need to be a change to the workflow to first save the images, and then to have a path to apply AI to these images whilst still allowing readers to view the processed images.

We are primarily concerned with the variability in image presentation that can affect AI. However, medical physics departments with a responsibility for ensuring quality in equipment should also be interested in this work. This work demonstrates differences in imaging system setup and provides a basis for advice to the imaging centres. It is well known that the imaging system setup can affect cancer detection [11,28,29,30]. Some of these factors affecting image quality are routinely tested by medical physics and screening units during quality control to ensure the quality of the images. However, generally, there is little testing undertaken routinely on image processing by Medical Physics departments. To reduce the variability of image appearance, the systems in a breast screening unit should be set up in the same way. In addition, staff should be monitoring the quality of images using clinical audit and ensuring that the image processing is adequate across all of the subsets of clinical images for different compressed breast thicknesses, glandularities and other features such as implants. This is not a simple process, but must be considered as a multidisciplinary process including radiologists, radiographers and physics staff. Useful information on the audit process of clinical images can be found in an EUREF report [31]. This work demonstrates that it is potentially valuable to audit the settings of clinical images, although it is not necessarily simple to extract this data from the Picture Archiving and Communication System (PACS) [32].

DICOM tags are described elsewhere [33], but here we provide useful background information on the DICOM header for mammography. We have shown the location of some of the useful tags, especially image processing. There are challenges highlighted here as data are stored in different locations between manufacturers. It is also difficult to interpret the outcomes; often, the information in the image processing tag is an array of letters and numbers, without a key to understand them.

A number of limitations must be noted: the DICOM header data were acquired by different medical physics groups across the UK, and although there is a protocol, there can be subtle differences in the acquisition methods. However, the acquisition method should have a minimal effect on the data in the DICOM tags used in this study, with the possible exception of the dose level on the GE systems, which could have been selected manually at the time of acquisition. We have visually compared a few examples of TORMAM images to see how the factors listed in this work can affect the image presentation. Though the image processing might not function optimally for this phantom, the images will still give the reader some indication of the possible differences in processed images. The DICOM tags were not always complete in the header, and we know from experience that even complete tags can contain errors [34,35]. The next step for our study will be to evaluate whether the differences in versions and image perception will lead to a difference in AI scores.

## 5. Conclusions

It is known that the perception of images may affect the decision of human or AI readers. This retrospective audit has shown the presence of a number of variations in the settings of mammographic equipment that are likely to affect the image characteristics and image appearance. For Hologic systems, we also showed that there are several different software versions in concurrent use at any one time; this is expected to be similar for other systems.

The datasets used to train AI systems may not closely reflect current clinical images in breast screening, as the AI algorithms would likely have been developed using older images obtained from different systems. The effectiveness of AI will be reliant on its generalisability to adapt to differences in setup. Therefore, there is a need for independent testing of the outcome and robustness of AI systems to ensure the expected result of AI when changes in image characteristics/appearance occur.

There are responsibilities on AI suppliers, physics services, mammographic equipment manufacturers, and breast screening units to manage the use of AI and ensure the outcomes of breast screening are not adversely affected by the setup of equipment.

## Figures and Tables

**Table 3:** Information on the updates associated with software updates for the Hologic.

Software Version	Software Update
1.5.1	Processing of implant images: contrast and brightness of images
1.9.0	Metal artefact algorithm improved. Skin line improvements
1.9.2	Processing applied to images with the 18 × 24 SmartCurve paddle has been updated
1.10.0.412	Higher dose for small breast (up to 50 mm breast thickness)
	Breast edge algorithm update reducing the white skinline (optional)
	Masking of implants implemented (optional)
1.10.25	Update the grid device
1.11	Update processing for smartcurve paddle
1.11.0.386	Implant brightness adapted

**Table 4:** Number of dose levels for Siemens (numbers in brackets are PRIME).

Dose Range	Inspiration	Revelation
<100%	4 (4)	1 (1)
100% (standard)	12 (0)	13 (0)
110% to 119%	7 (0)	3 (3)
120% to 129%	12 (0)	5 (0)
130% to 140%	0 (0)	10 (0)
150%	8 (0)	0 (0)

**Table 5:** Variability of Hologic image processing subtags by AWS software version group.

AWS Software Versions	Total Images	No. of Subtags in (0019x, 1062x) Tag	No. of Differences in Values of Subtags
1.3.0.116 to 1.5.2.3	18	11	3
1.6.2.54 to 1.6.5.1	9	51	1
1.7.1.37 to 1.7.4.5	96	122	3
1.8.3.63 to 1.9.1.8	154	125	5
1.10.0.412	39	134	3
1.11.0.386 to 1.11.1.75	92	145	5

## Data Availability

The authors do not own the data. The dataset with sharing permission has been included as Supplementary Materials.

## Supplementary Materials

The following supporting information can be downloaded at: https://www.mdpi.com/article/10.3390/diagnostics16121842/s1.

## Abbreviations

The following abbreviations are used in this manuscript:

AEC	Automatic Exposure Control
AI	Artificial Intelligence
AWS	Acquisition Work Station
DICOM	Digital Imaging and Communication in Medicine
GE	General Electric
Ho	Hologic
HTC	High Transmission Cellular
MPD	Medical Physics Department
NHS	National Health Service
NHSBSP	National Health Service Breast Screening Programme
PACS	Picture Archiving and Communication System
PRIME	Progressive Reconstruction, Intelligently Minimising Exposure
QC	Quality control
RRPS	Regional Radiation Protection Service
Sm	Siemens
UK	United Kingdom
